# Supplementary material for: Plasma Proteins Associated with COVID-19 Severity in Puerto Rico
Source: Int J Mol Sci. 2024 May 16;25(10):5426. doi: 10.3390/ijms25105426 (PMC11121485; doi:10.3390/ijms25105426)
Supplement: Supplementary file 1 [file ijms-25-05426-s001.zip › Supplementary Figures.pdf]

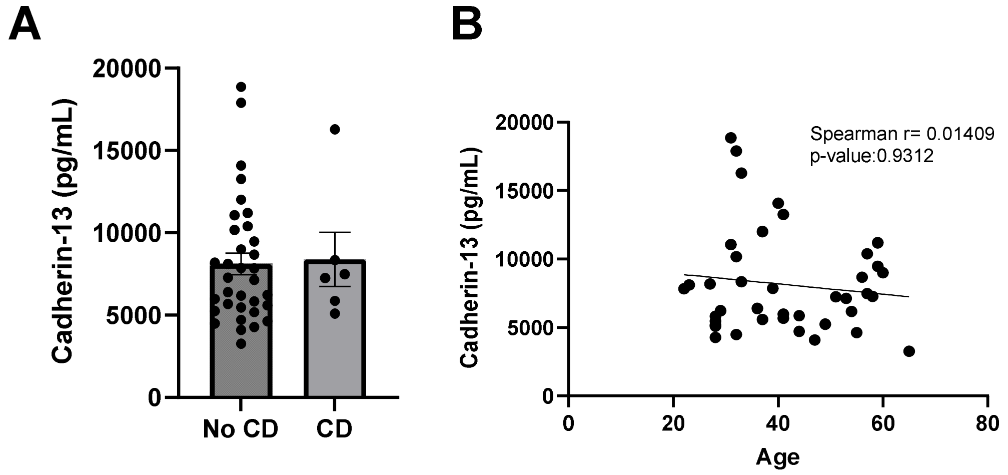

Figure S1. Association of Cardiovascular Disease (CD) and Age with Cadherin-13 levels. Plasma samples from unvaccinated healthy controls (*n*=8) and COVID-19 positive patients stratified by severity (Mild: *n*=12, Moderate: *n*=12, and Severe: *n*=8) were randomly selected for validation using a human Cadherin-13 ELISA. A. Comparison of Cadherin-13 levels between participants with No CD (*n*=34) and CD (*n*=6), independently of COVID-19 positivity status. The mean +/- SEM is shown. B. Correlation of age vs cadherin-13 levels in all participants included in the Cadherin-13 ELISA (*n*=40), independent of COVID-19 positivity status.

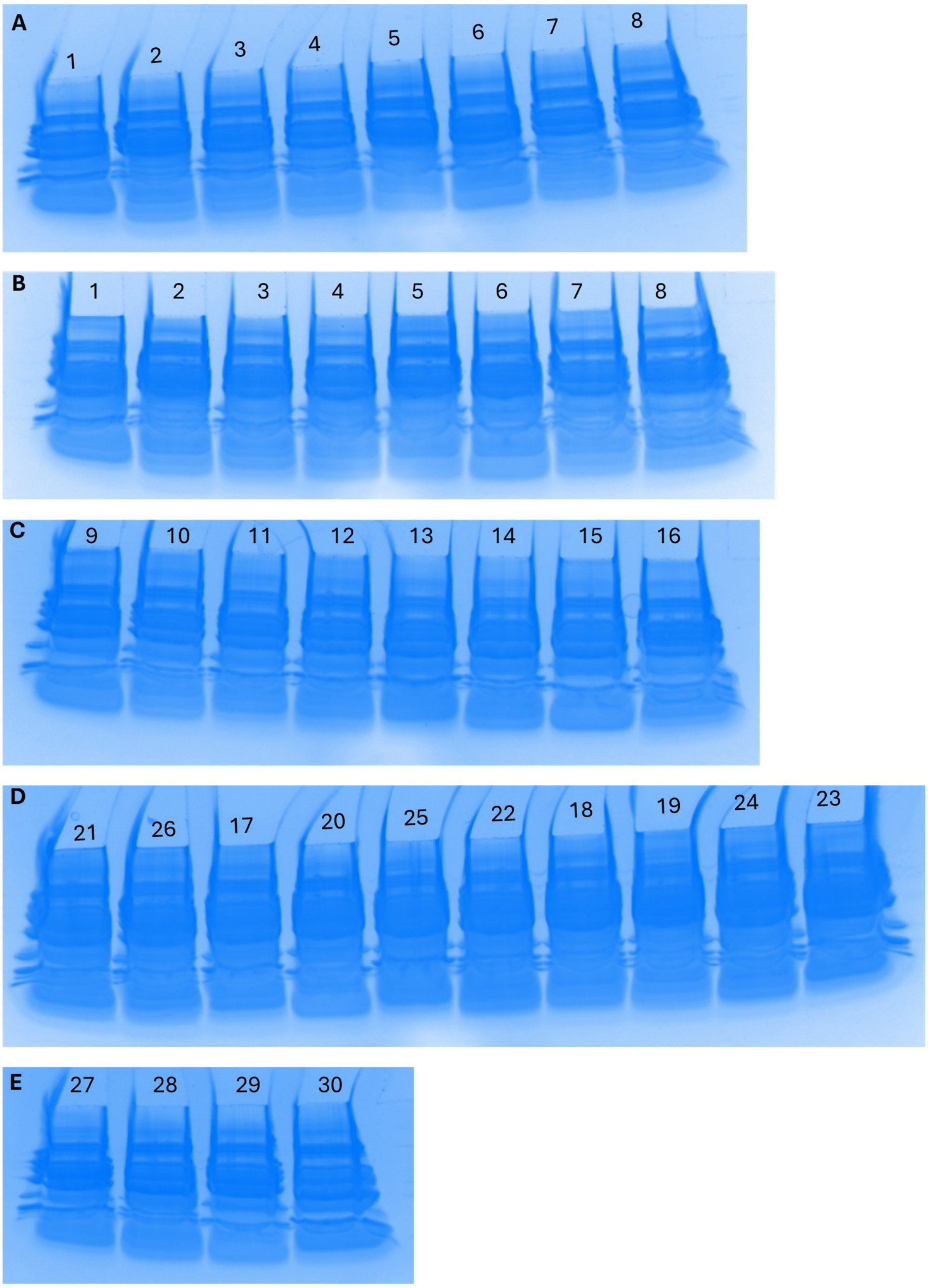

Figure S2. Coomassie-Stained Gels. Short-run electrophoresis at 150V for 15min with 100ug of total protein (*n*=30) was performed and gels were stained with Coomassie to cut out proteome bands. COVID-19 negative samples (*n*=8) were run in duplicates (A and B) to have sufficient material for an internal control for the TMT. Gel C contains Mild COVID-19 positive patients (*n*=8). Gel D contains Moderate COVID-19 positive patients (*n*=10). Gel E contains Severe COVI-19 positive patients (*n*=4). Each lane is numbered with the corresponding participant's number according to Table S9.
