# Supplementary material for: Plasma Proteins Associated with COVID-19 Severity in Puerto Rico
Source: Int J Mol Sci. 2024 May 16;25(10):5426. doi: 10.3390/ijms25105426 (PMC11121485; doi:10.3390/ijms25105426)
Supplement: Supplementary file 1 [file ijms-25-05426-s001.zip › Table S10-TMT Labeling Design.pdf]

A. TMT - 1		
**TMT Label**	**Sample ID**	**Category**
126	***Aliquot #1***	***Internal Pool (All Controls)***
127N	1	COVID-19 Negative
127C	2	COVD-19 Negative
128N	3	COVID-19 Negative
128C	9	COVID-19 Mild
129N	10	COVID-19 Mild
129C	11	COVID-19 Mild
130N	17	COVID-19 Moderate
130C	18	COVID-19 Moderate
131N	19	COVID-19 Moderate
131C	27	COVID-19 Severe
**B. TMT - 2**		
**TMT Label**	**Sample ID**	**Category**
126	***Aliquot #2***	***Internal Pool (All Controls)***
127N	28	COVID-19 Severe
127C	4	COVID-19 Negative
128N	5	COVID-19 Negative
128C	6	COVID-19 Negative
129N	12	COVID-19 Mild
129C	13	COVID-19 Mild
130N	14	COVID-19 Mild
130C	20	COVID-19 Moderate
131N	21	COVID-19 Moderate
131C	22	COVID-19 Moderate
**C. TMT - 3**		
**TMT Label**	**Sample ID**	**Category**
126	***Aliquot #3***	***Internal Pool (All Controls)***
127N	23	COVID-19 Moderate
127C	24	COVID-19 Moderate
128N	25	COVID-19 Moderate
128C	26	COVID-19 Moderate
129N	29	COVID-19 Severe
129C	30	COVID-19 Severe
130N	15	COVID-19 Mild
130C	16	COVID-19 Mild
131N	7	COVID-19 Negative
131C	8	COVID-19 Negative

Table S10. TMT Labeling Design. Plasma aliquoted samples from 22 (n = 22) unvaccinated COVID-19 patients stratified by disease severity and negative controls (n=8) were randomly selected for quantitative proteomics studies. Samples were assayed in three 11-plex TMT kits

as depicted in A, B, and C. These participants were controlled by age, sex, and comorbidities and numbered (Sample ID) according to Table S9. COVID-19 patients were stratified as follows: mild (n = 8), moderate (n = 10), and severe (n = 4) disease. Controls included eight (n=8) COVID-19 negative participants. To obtain the Internal Pool an additional aliquot of all COVID-19 negative control samples that are mixed in one tube; then the volume in this tube is divided into three equal aliquots that are included in each of the three kits (one aliquot per kit) to enable data comparison between all kits. The samples included in the Internal Pool come from the following COVID-19 negative participants**:** 1, 2, 3, 4, 5, 6, 7, and 8.
