# Supplementary material for: Plasma Proteins Associated with COVID-19 Severity in Puerto Rico
Source: Int J Mol Sci. 2024 May 16;25(10):5426. doi: 10.3390/ijms25105426 (PMC11121485; doi:10.3390/ijms25105426)
Supplement: Supplementary file 1 [file ijms-25-05426-s001.zip › Table S1-Proteomics studies of COVID-19 severity.pdf]

Table S1. Plasma and serum proteomics studies and COVID-19 disease severity in the world

Study	Population (n)	Gender	Ages	Quantitative Proteomics method	Proteins associated to severity
1.Arunachalm et al., 2020[65]	N=76 COVID-19 patients from Hong Kong<br><br>N=69 Healthy individuals from Atlanta, Georgia, United States.	China 58% M<br><br>Atlanta 55% M	China 18-80 y/o Median-55y/o<br><br>Atlanta 25-94 y/o Median 56y/o	Multiplex analysis of cytokines in the plasma of COVID-19 patients using Olink Proteomics. Patients were classified based on COVID-19 severity.	Increased expression with disease severity:<br><br>*TNFSF14* [LIGHT, a ligand of lymphotoxin B receptor highly expressed in human lung fibroblasts and implicated in lung tissue fibrosis and remodeling and inflammation. *TNFSF14* is distinctively enhanced in the plasma of COVID-19–infected individuals but not in cases of other related pulmonary infections.<br><br>EN-RAGE (*S100A12*), a biomarker of pulmonary injury that is implicated in pathogenesis of sepsis-induced ARDS.<br><br>Oncostatin M (*OSM*), a regulator of IL-6.<br><br>*TNFSF14*, EN-RAGE, and *OSM* were validated by ELISA and found increased with disease severity.
2. Ciccosanti et al., 2022[23]	N= 48 total Rome, Italy N=32 COVID-19<br><br>N=16 Healthy Donors	62.5% males	Median<br><br>Healthy Donors: n=43 y/o<br><br>non-ICU n=67.5 y/o<br><br>ICU n=65 y/o	Mass spectrometry (MS), Liquid chromatography (LC)-(MC) and ELISA using Thermo Scientific Q Exactive Plus.<br><br>Validation of plasma protein levels by Human ELISA	Increased in all COVID-19 patients but at higher levels in ICU/F patients: Proteins playing a role in acute inflammatory response (*CRP*, *ORM1*, *ORM2*, *SAA1*, SAA2, *S100A8*, *S100A9*, Serpin A3). Confirmed by ELISA that higher levels of the neutrophil granule proteins *DEFA3* and *LCN2* in COVID-19 patients requiring ICU admission when compared to non-ICU and healthy donors. Increased levels of histone *H4*, *MPO*, *LCN2*, *PGLYRP1*and *DEFA3* support the observation that excessive NET formation occurs in severe COVID-19 patients.
3. D'Alessandro et al., 2020[31]	Columbia, USA N= 49<br><br>COVID-19+: N=33<br><br>Healthy: N=16	75% M	median age 56 y/o	ELISA and Nano Ultra-High-Pressure Liquid Chromatography Tandem Mass Spectrometry (MS/MS) and Metabolomics	Stratified by IL- 6 levels, significant increase in serum levels of: SERPINs and carboxypeptidases (*CPB2*/*TAFI*) in the coagulation/fibrinolytic cascade, including *SERPINA1*, *SERPINA3*, *SERPINF2*. Increased circulating levels of several coagulation factors, specifically Factor 5, 7, and 10 in patients with the highest IL-6 levels. IL-6 stimulated the increase in the levels of matrix metal- loproteinases (MMPs), matrilysin (*MMP7*), and stromelysin-1 (*MMP3*), which can cleave subclasses of IgG And decreased levels of: Factor XIIIb and gelsolin

4. Demichev et al., 2021[26]	Berlin, Germany N=139	68% M	50-71 y/o<br><br>41% older than 65	SWATH/DIA-MS	High levels with poor prognosis: *AGT*: Angiotensinogen, B2M: Beta-2-microglobulin, *C1R*: Complement *C1*, *CFD*: Complement Factor D, *CRP*: C-reactive protein, *CST3*: Cystatin C, and *SERPINA3*: Alpha-1-antichymotrypsin<br><br><u>Low levels</u> with poor prognosis: AHSG: Alpha-2-HS glycoprotein (Fetuin-A) attenuate macrophage activation and neutrophil degranulation; *HRG*: Histidine-rich glycoprotein: regulator of inflammation and immune response, *ITIH2*: Inter-alpha-trypsin inhibitor heavy chain H2: has matrix-stabilizing and immunomodulatory effects *PLG*: Plasminogen, Plasmin: Mediator of fibrinolysis, neutrophil attenuation, macrophage efferocytosis and polarization from M1 to M2 phenotype.<br><br><u>Downregulated</u> in severe COVID-19: The coagulation cascade with known acute phase activity, such as fibrinogen, and many complement factors. Proteins indicative of inflammatory response (e.g., *ORM1*, *SERPINA1* and *SERPINA3*, *SAA1*, *SAA2* and markers of inflammation, such as *CRP* or IL-6. Extracellular matrix (ECM) proteins, such as ECM1, LUM, and immunoregulatory factors and proteins involved in lipid metabolism (*APOC1*, *APOD*, *APOM*, *GPLD1*, and *PON1*), and APPs.
5.Kimura et al., 2021[66]	Japan n=10	Not specifie d	Not specified	(DIA-MS), LC mass spectrometry, and ELISA	Chitinase-3-like protein 1 (*CHI3L1*) and insulin-like growth factor-binding protein acid labile subunit (*IGFALS*), correlated with adverse prognosis in severe COVID-19 patients. Elevated *CHI3L1* levels with severe disease and adverse prognosis. Reduction of *IGFALS* levels in severe COVID-19
6.Messner et al., 2020[22]	Germany n=199 controls from Scotland and 34 COVID patients.	Not specifie d	Not specified	SWATH/DIA-MS	Detected activation of both the classical complement pathway (*C1R*, *C1S*, and *C8A*) and the alternative pathway factor B (*CFB*), complement modulators: factors I (*CFI*) and H (*CFH*). Pro-inflammatory signaling both upstream and down- stream of interleukin (IL)-6 were differentially expressed with disease severity. <u>Upregulated</u> inter-a-trypsin inhibitor heavy chain 4 (*ITIH4*) (extracellular matrix organization, alpha-1B-glycoprotein

					(*A1BG*), beta and gamma-1 actin (*ACTB;ACTG1*), monocyte differentiation antigen and lipopolysaccharide coreceptor *CD14*, lipopolysaccharide-binding protein (*LBP*), galectin 3-binding protein (*LGALS3BP*), leucine-rich alpha-2-glycoprotein (*LRG1*), haptoglobin (*HP*), protein Z-dependent protease inhibitor (*SERPINA10*), C-reactive protein (*CRP*) serum amyloid proteins *SAA1* and *SAA2* Downregulated: gelsolin (*GSN*), and transferrin (*TF*), albumin (*ALB*) (downregulated, *APOA1*, *APOC1* (downregulated).
7.Nuñez et al., 2022[27]	Spain n=72 (Discovery) n=84 (Validation)	Non Hospitalized <60 y/o (38% M), 60-80 y/o (50% M)<br><br>Hospitalized <60 y/o (56% M), 60-80 y/o (70% M) >80 y/o (33% M)	Non Hospitalized <60, and 60-80 y/o<br><br>Hospitalized <60, 60-80 and >80 y/o	10-plex tandem mass tags (TMT). Q Exactive HF mass spectrometer (Thermo). Protein quantification and statistical and systems biology analysis were performed using SanXoT software package. Validation: Targeted Proteomics. SRM method and Skyline software. Selected peptides were between 7–25 amino acids in length.	A panel of 53 proteins that participate in several functions such as acute-phase response and inflammation, blood coagulation, cell adhesion, complement cascade, endocytosis, immune response, oxidative stress, and tissue injury, have been correlated with patient severity. Eighteen protein candidates were further validated by targeted proteomics in an independent cohort of 84 patients including a group of individuals that had satisfactorily resolved SARS-CoV-2 infection. Remarkably, all protein alterations were normalized 100 days after leaving the hospital, *CRP*, *SAA1* and *ALB*, ITIHs, *CD14*, *LBP* and *LRG1*, *GSN*, *ACTB* and *CST3*. *APOA1*, *APOA2* and *APOL1* are components of HDL.
8.Pagani et al., 2023[24]	Italy n=43<br><br>33 mild and 10 Severe WHO Score 4/5	53.4% M	Median56 y/o	An untargeted and label-free proteomic approach has been applied to plasma samples.	MILD and hospitalized patients in need of oxygen support therapy (SEVERE) highlighted 29 proteins emerged as differentially expressed: 12 overexpressed in MILDs and 17 in SEVEREs. Fetuin-A, Ig lambda-2chain-C-region, Vitronectin) that can discriminate between MILD and SEVEREs (*SAA1/2*, *CRP*, *HP*, *LRG1*) and in MILDs (*GSN*, *HRG*).
9.Palma Medina et al., 2023[67]	Sweden N= 33 total (n = 17, severe COVID-19) (n=10, moderate COVID-19).	77% M	41-63 y/o	Olink 96-targets panels were used for inflammation. Used machine learning to pinpoint a set of bio- markers that could accurately	Seven proteins, i.e., *TRIM21*, *CASP8*, *NBN*, *FOXO1*, *PIK3AP1*, *PTN*, and *BID*, had higher average variable importance for the models. On average, the models had high accuracy in differentiating COVID-19 from CAP. Among these proteins, *KRT19*, *TOP2B*, *AREG*, *HGF*, *CKAP4*, *ITGB6*, and *NCF2* had a higher expression (> two-fold)

	Samples from age- and sex-matched SARS- CoV-2 IgG seronegative healthy volunteers (n=16)			discriminate COVID-19 from CAP-sepsis.	in plasma samples of severe compared to moderate COVID-19 patients, whereas *CLEC4C* and *LTA* were among the few proteins that were expressed at lower levels in severe patients.
10.Park et al., 2020[20]	Korea n=8<br>3 non severe<br>5 severe	50% M	26-76 y/o	Label-free LC/MS/MS Statistical tests with stringent criteria (Student's t-test, p value < 0.05, and ∣fold-change∣> 1.5).	A total of 91 out of 1222 quantified proteins were differentially expressed depending on the severity of COVID- 19. Found 76 proteins, previously not reported. 7 DEPs (*IGFBP3*, *ITIH4*, *SERPINA3*, *ORM1*, *VWF*, *SERPING1*, and *LBP*) were commonly identified with disease severity. All these proteins except *IGFBP3* showed identical trends of expression in different patient cohorts. These 6 proteins may constitute a reliable blood marker for classification of COVID-19 severity.
11.Roh et al.,2022[68]	Boston, Massachusetts, USA N=80<br><br>COVID-19+: N=54<br><br>Healthy: N=26	41% M	Mean age: 64 y/o	An aptamer-based proteomics platform (version 4, Somalogic) was used to measure relative levels of 4,996 analytes, corresponding to 4,730 distinct human proteins.	Among 4,996 analytes measured, *ADAMTS13*, the vWF-cleaving protease whose loss-of-function causes microvascular thrombosis, displays the most significant inverse association with myocardial injury in COVID-19. Increased Activin/TGFb signaling is strongly associated with the heart failure biomarker NT-proBNP in COVID-19. *SASP*, a marker of biological aging, is the dominant process associated with cardiac involvement and COVID-19 severity.
12.Shen et al., 2020[21]	China N=118<br><br>n=65 COVID<br><br>Controls n=53	COVID: 41% M<br><br>Controls: 75% M	COVID: ages 18-77 y/o<br><br>Control: ages 23-67 y/o	Digested peptides were cleaned-up with SOLAm, and labeled with TMTpro 16plex label reagents. The TMT samples were fractionated using a nanoflow DIONEX UltiMate 3000 RSLCnano System Targeted proteomics x validation. TripleTOF 6600 system (SCIEX, CA, USA) was applied	Upregulation of *SAA1*, *SAA2*, *SAA4*, *CRP*, *SERPINA3*, *SAP*, Vascular cell adhesion protein 1 (VCAM-1), which helps to regulate trans endothelial migration of leukocytes by stimulating production of reactive oxygen species (ROS), was upregulated with severe COVID. Elevation of glucose, glucuronate, bilirubin degradation product, and four bile acid derivatives was found in metabolomic data from severe patients indicating reduced liver detoxification.

				for MRM-HR experiment.	
13.Suvarna et al., 2021a[25]	India<br>N=71<br><br>(20 COVID-19 negative, 18 COVID-19 non-severe, and 33 severe)	Controls:<br>70% M, 30% F<br><br>non-severe COVID:<br>61.1% M,<br>38.9% F<br><br>Severe COVID:<br>72.7% M,<br>27.7% F	MedianControl: 39 y/o<br><br>Non-severe COVID: 59y/o<br><br>Severe: 56 y/o	The MS analysis was performed using an Orbitrap Fusion Tribrid Mass Spectrometer. Label-free quantification (LFQ) using mass-spectrometry analysis identified 38 differentially expressed proteins in severe COVID-19 patients when compared with non-severe.	Of the 1200 proteins detected in the patient plasma, 38 were identified to be differentially expressed between non-severe and severe groups. Pathways related to peptidase activity, regulated exocytosis, blood coagulation complement activation, leukocyte activation involved in immune response, and response to glucocorticoid biological processes in severe cases of SARS-CoV-2 infection. They used machine learning approaches to identify the classifiers of patients with non-severe and severe COVID-19. Upregulated in severe: kallistatin (*SERPINA4*), serum amyloid P-component (*APCS*), protein S100-A8 (*S100A8*), fibrinogen gamma chain (*FGG*), corticosteroid-binding globulin (*SERPINA6*), and alpha- 1-antichymotrypsin (*SERPINA3*). Downregulated in severe such as complement factor D (*CFD*), monocyte differentiation antigen (*CD14*), complement component C8 alpha chain (*C8A*), apolipoprotein (*LPA*), and apolipoprotein M (*APOM*). *APOB*, *SERPINA3*, and *FGG* validated with SVM model to distinguish severe and non-severe samples.
14.Wang et al., 2022[69]	Germany<br><br>Severe patients with COVID-19 hospital-ised before (n=30) and after dexamethas one, (n=164)	n=39 F<br><br>n=125 M	Ages 51-69 y/o	Selected a panel of 50 peptides, derived from 30 proteins, whose functions have been associated with COVID-19 using Agilent 6495C mass spectrometer, coupled to an Agilent 1290 Infinity II UHPLC system.<br><br>Validated a scalable proteomic panel assay on a SCIEX 7500 mass spectrometer coupled to an ExionLC AD	Proteins that predicted COVID severity: *SERPINC1* = Antithrombin-III, *C3* = Complement C3, *APOB* = Apolipoprotein B, *SERPING1* = C1-inhibitor, *CST3* = cystatin C, *VWF* = von Willebrand factor, *CRP* = C-reactive protein, *PLG* = plasminogen, *KLKB1* = plasma kallikrein, *LYZ* = lysozyme,and *APOA1* = Apolipoprotein A. From the final selected panel, 18/30 proteins are associated with remaining time in hospital, 22/30 with disease severity, and 6/30 are prognostic of future worsening. Six additional peptides included were prognostic for remaining time in hospital (*PRG4*, *C3*, *EFEMP1*, *ORM2*, *FCGR3A*, *AFM*, *IGHVs*).

				UHPLC system (SCIEX, UK).	
15.Wang et al., 2023[30]	Missouri, USA<br>n=482<br>(332 COVID-19 patients and 150 controls)	COVID-19<br>195 (58.7%) M<br><br>Control<br>85 (56.6%) M	COVID-19<br>59.7 ± 16.5 y/o<br><br>Control<br>65.7 ± 14.0 y/o	Pursued a three-stage study design (discovery, replication, and meta-analysis). randomization (MR) (STAR Methods).	Higher troponin T, *ANGL4*, *FURIN* levels in COVID-19 infection and ventilation. Lower *ATP1B1* levels were found in COVID-19 infection and ventilation. Proteins that are in AD pathway and associated with poor COVID-19 outcomes include *APP*, *NFL*, *EPHA5*, *TMEM106B*, *MAPT*, and *GFAP* among others. Proteins that are part of the coronary artery disease pathway include troponin T, *ANGL4*, *FURIN*, *AT1B1*, and *TGFB1*.
16.Asare-Werehene et al., 2022[32]	Japan<br>N=40<br><br>COVID-19+:<br>N=28<br><br>Negative Controls:<br>N=12	COVID-19+:<br>6 F, 22 M<br><br>Control:<br>7 F, 5 M	Negative Control:<br>Mean=41.67 y/o<br><br>COVID-19+:63-93 y/o	Blood samples were longitudinally collected from hospitalized COVID-19 patients and negative controls to measure plasma Gelsolin (pGSN) in mg/mL, cytokines and anti-SARS-CoV-2 spike antibodies to develop a prognostic platform.	pGSN levels were significantly reduced in COVID-19 patients compared to healthy individuals. pGSN levels combined with plasma IL-6, IP-10 and M-CSF significantly distinguished COVID-19 patients from healthy individuals. pGSN and anti-spike IgG titers together strongly predict COVID-19 severity and death, the combination of pGSN and IL-6 was significant predictor of milder disease and favorable outcomes
17.Zoodsma et al., 2022[70]	Netherlands<br><u>Cohort 1(Breda):</u><br>n= 59 COVID-19 ICU<br>n= 148 COVID-19 non-ICU<br><br>Cohort 2(Nijmegen):<br>n= 38 COVID-19 ICU<br>n= 106 COVID-19 non-ICU<br><br><u>Cohort</u>	<u>Cohort 1:</u><br>ICU: 45M/14F<br>Non-ICU: 88M/60F<br><br>Cohort 2:<br>ICU: 28M/9F<br>Non-ICU: 69M/37F<br><br><u>Cohort 3:</u><br>Post-	<u>Cohort 1:</u><br>ICU (mean age: 67 ± 9 y/o) and non-ICU (mean age: 70 ± 12 y/o)<br><br><u>Cohort 2</u>:<br>ICU (mean age: 64 ± 12 y/o)<br>non-ICU (mean age: 63 ± 14 y/o)	The multiplex proximity extension assay (PEA) from Olink Proteomics AB was used to quantify circulating proteins in plasma. Upon linking, the probe sequence hybridizes and is extended by DNA polymerase. The resulting sequence acts as a unique identifier for the protein and is quantified by a real-time polymerase chain reaction. Proteins are expressed as	350 hospitalized COVID-19 patients, 186 post-COVID-19 individuals, and 61 healthy individuals from 3 independent cohorts. ICU patients had increased hepatocyte growth factor, *CCL20* and *MMP10* In contrast. ICU patients in both cohorts had lower stem cell factor (SCF), Delta and Notch-like epidermal growth factor-related receptor (*DNER*), and TNF-related weak inducer of apoptosis. TRAIL, which was significantly downregulated in ICU, but not in non-ICU patients. MMPs, involved in the proteolytic degradation and maintenance of the ECM, as well as ECM-receptor interactions were found to be enhanced in acute COVID-19

	3(Hannover):<br>n= 187 post-COVID-19<br>n= 61 healthy individuals	covid: 99M/ 87F<br>Healthy: 36M/ 25F	Cohort 3: post-COVID (mean age: 43 ± 12 y/o)<br><br>Control(mean age: 46 ± 14 y/o)	normalized protein expression (NPX) values, a relative value on a log2 scale.	
18. Alghanem et al., 2023[46]	Saudi Arabia<br><br>N=25<br><br>Healthy (n=5), Mild (n = 5), Moderate (n = 5), Severe (n = 5), and Dead (n = 5)	Not specified	Not specified	iTRAQ 4plex and NanoLC-MS/MS	Downregulation of Fetuin-A, Tetranectin (*TN*), and Paraoxonase-1 (*PON1*) correlates with COVID-19 disease severity.
19. Byeon et al., 2022[55]	USA<br><br>N=637<br><br>Controls (n=182)<br><br>Outpatients (n=183)<br>Severe COVID-19 (n=139)<br>critical COVID-19 (n=133)	Controls :54% F<br><br>Outpatients:61% F<br><br>Severe: 50% F<br><br>Critical: 39% F	Median:<br><br>Controls: 58·8<br><br>Outpatients: 47·9<br><br>Severe: 62·1<br><br>Critical: 63·1	Olink Explore 1536 panel assay (Olink Proteomics) and LC-MS-MS. Machine Learning	The following proteins were elevated in plasma and showed a positive association with disease severity: *SIGLEC5*, *CLEC6A*, *CCL7*, *AZU1*, *CAPG*, *LILRA5*, *CXCL9*, *CXCL10*, *CXCL11*, *LAG3*, *LGALS9*, *LTA4H*, *VSIG4*, *TNFRSF10A*, *FAS*, *SDC1*, *MDK*, *LRP1*, *MMP7*, *VEGFA*, *ANGPTL1*, *EPHB4*, and *DDAH1*<br><br>The following proteins were downregulated in plasma or showed a negative association with disease severity: *ICOSLG*, *TNFSF10*, *TNTFSF11*, *COMP*, *CD1C*, *CRTAC1*, *KLK13*
20. Alaiya et al., 2021[28]	Saudi Arabia<br><br>N=132<br><br>Controls (n=10), Asymptomatic (n=29), Mild (n=47), Moderate (n=24), and	41.73% F	Median: 47	Label-free quantitative proteomics by LC-MS-MS	They found 193 differentially expressed proteins across different grades of COVID-19. They highlighted proteins that have been previously identified by Messner et al 2020, which included the following proteins associated with severe disease: *CRP*, *SAA2*, *FGG*, *ACTB*, *SAA1*, *LRG1*, *APOC1*, *HP*, *ITIH4*, *LBP*, *C8A*, *ALB*, *TF*, *CFI*, *CFHR1*, and *GSN*.

	Severe (n=22)				
21. Li et al., 2021[71]	USA<br><br>N=300<br><br>Aviremic (n=247), Viremic (n=53)	48% F	Range: <50 to ≥65	Proximity Extension Assay using the Olink platform.	Among the several proteins identified, they highlighted the elevation of SARS-CoV-2 entry factors *ACE2*, *CTSL*, and *FURIN*.
22. Suvarna et al., 2021b[72]	India<br><br>N=13 (proteomics)<br><br>Non-severe (n=7), Severe (n=6)	100% M	Range: 34-77	Orbitrap Fusion LC–MS/MS-based label-free proteomics	Upregulated proteins: *RAP1B*, *IGFALS*, *PDIA3*, *HSPA8*, *PDLIM1*, *PFN1*, *PTGDS*, *SERPINA10*<br><br>Downregulated proteins: *TGFBI*, *LGALS3BP*
23. Messner et al., 2021[33]	Germany<br><br>healthy patients (n = 15), Mild (n = 5-10), Severe (n = 4-7), and critical (n= 8-13)	46.6% F	Range: 21-86	LC-MS-MS with Scanning SWATH	Upregulated: *CFI*, *ITIH4*, *C1QC*, and *PROS1*.<br><br>Downregulated: *GSN*, *A2M*, *HPX*, *IGHG2*, *IGKV4-1*, *PON1*, *SERPINA7*, *SERPINF2*, *TMEM198*, and *TTR*.
24. Duijvelaar et al., 2024[73]	Netherlands<br><br>N = 318<br><br>Non-critical illness (n = 249), critical illness (n = 69)	Non-critical: 32.9% F<br><br>Critical: 20.3% F	Mean:<br><br>Non-critical: 63<br><br>Critical: 69.2	Proteomics using aptamer assays by SomaLogic, Inc	The most significant proteins associated with clinical illness included integrins, ICAMs, *IBSP*, RGMa, collagens, biglycan, thrombospondin-2, nidogen-1, *LAMA4*, *FGF4*, *FGF8*, TGF-β1, TGF-β3, and *BMP10*.
25. Villar et al., 2021[74]	Spain<br><br>N= 95<br><br>Asymptomatic (n = 16), recovered (hospital discharge; n = 26), nonsevere	F/M ratio:<br><br>Healthy: 1.1<br>Asymptomatic: 0.9<br>Nonsevere	Mean:<br><br>Healthy: 41.4<br>Asymptomatic: 43.4<br>Nonsevere hospitalized: 73.7	SWATH-MS	Carboxypeptidase B2 (*CBP2*) was validated by ELISA as a protein associated with severity.

	(hospitalized; n = 28), and severe (ICU; n = 25)	hospitalized: 0.8<br>Recovered: 0.9<br>Severe: 0.7	Recovered: 61<br>Severe: 57.2		
26. Bu et al., 2024[11]	Canada<br><br>N=1,211<br><br>COVID-19+ (n=926) and hospitalized negative controls (n=285)	COVID-19+: 46% F<br><br>Controls: 47% F	Median:<br><br>COVID-19+: 60<br><br>Controls: 56	SOMAscan proteomics assay	Identified and validated by ELISA increased GDF-15 levels in plasma with increase COVID-19 severity.
27. Harriott & Ryan, 2024[12]	USA<br><br>N=70<br><br>Severe (n=22), Moderate, and (n=22) Mild (n = 10)	39% F	Range: <18 to >65	Olink proximity extension assays	They found several proteins associated with severity and highlighted the increased expression of mesothelin (*MSLN*) in severe patients as this protein has not been reported in COVID-19 severity studies previously.
