# Supplementary material for: Plasma Proteins Associated with COVID-19 Severity in Puerto Rico
Source: Int J Mol Sci. 2024 May 16;25(10):5426. doi: 10.3390/ijms25105426 (PMC11121485; doi:10.3390/ijms25105426)
Supplement: Supplementary file 1 [file ijms-25-05426-s001.zip › Table S9-Demographics of Patients included in TMT.pdf]

Table S9. Characteristics of participants included in TMT Labeling Proteomics				
**COVID-19 Negative (*n* = 8)**	**Age**	**Sex (F/M)**	**Comorbidities**	**Clinical Symptoms**
1	32	M	Prostate cancer	N/A
2	28	F	N/A	N/A
3	56	F	N/A	N/A
4	22	F	N/A	N/A
5	59	M	N/A	N/A
6	49	F	N/A	N/A
7	32	F	N/A	N/A
8	31	M	N/A	N/A
**COVID-19 Positive Mild (*n* = 8)**				
9	40	F	Oral herpes	Cough, headaches, muscle ache, loss of smell, loss of taste
10	28	F	N/A	Cough, headache
11	36	M	N/A	Loss of smell, cough, fever, headache, muscle ache
12	55	F	Autoinmune disease, diabetes, high blood pressure, kidney disease	Cough, shortness of breath, headache
13	41	M	N/A	Loss of smell, loss of taste, muscle ache, fever, cough
14	32	M	HIV/AIDS	Fever, headache
15	47	F	N/A	Loss of smell, loss of taste, cough
16	37	M	N/A	Muscle ache, cough, fever, headache
**COVID-19 Positive Moderate (*n* = 10)**				
17	39	F	Oral herpes	Cough, fever, Shortness of breath, muscle ache, headache, diarrhea, loss of taste, loss of smell
18	41	M	N/A	Loss of smell, loss of taste, muscle ache, fever, headache, sore throat, abdominal pain
19	37	M	N/A	Loss of smell, loss of taste, muscle ache, cough, fever, sore throat, diarrhea, nausa, fatigue
20	31	F	Autoinmune disease, high blood pressure, kidney disease	Loss of smell, loss of taste, muscle ache, cough, fever, headache, chest pain
21	27	F	N/A	Loss of smell, loss of taste, muscle ache, headache, chest pain, nausea,
22	28	M	Autoinmune disease	Muscle ache, cough, shortness of breath, fever, headache, chest pain
23	51	M	Cardiovascular disease,	Loss of smell, loss of taste, cough, fever, shortness of breath, headache, muscle ache
24	33	M	Cardiovascular disease, lung disease	Shortness of breath. Required an oxygen mask without the need for hospitalization.
25	58	F	N/A	Loss of smell, loss of taste, cough, fever, muscle ache, shortness of breath, headache
26	28	F	N/A	Loss of smell, loss of taste, muscle ache, fatigue, sore throat, diarrhea

**COVID-19 Positive Severe (*n* = 4)**				
27	54	F	Cancer	Cough, fever, shortness of breath, headache, diarrea, nausea, abdominal pain
28	47	M	N/A	Cough, fever, fatigue
29	65	F	N/A	Muscle ache, shortness of breath, chest pain, fatigue, sore throat, diarrhea
30	60	M	Diabetes, high blood pressure	Headache , diarrhea
**p-value**	**0.0790**	**0.9467**	**0.3785**	
